# Human Adaptive Behavior in Common Pool Resource Systems

**DOI:** 10.1371/journal.pone.0052763

**Published:** 2012-12-28

**Authors:** Gunnar Brandt, Agostino Merico, Björn Vollan, Achim Schlüter

**Affiliations:** 1 Systems Ecology, Leibniz Center for Tropical Marine Ecology, Bremen, Germany; 2 School of Engineering and Science, Jacobs University, Bremen, Germany; 3 Institutional & Behavioural Economics, Leibniz Center for Tropical Marine Ecology, Bremen, Germany; 4 Institute of Public Finance, University of Innsbruck, Innsbruck, Austria; 5 School of Humanities and Social Sciences, Jacobs University, Bremen, Germany; Universidad Carlos III de Madrid, Spain

## Abstract

Overexploitation of common-pool resources, resulting from uncooperative harvest behavior, is a major problem in many social-ecological systems. Feedbacks between user behavior and resource productivity induce non-linear dynamics in the harvest and the resource stock that complicate the understanding and the prediction of the co-evolutionary system. With an adaptive model constrained by data from a behavioral economic experiment, we show that users’ expectations of future pay-offs vary as a result of the previous harvest experience, the time-horizon, and the ability to communicate. In our model, harvest behavior is a trait that adjusts to continuously changing potential returns according to a trade-off between the users’ current harvest and the discounted future productivity of the resource. Given a maximum discount factor, which quantifies the users’ perception of future pay-offs, the temporal dynamics of harvest behavior and ecological resource can be predicted. Our results reveal a non-linear relation between the previous harvest and current discount rates, which is most sensitive around a reference harvest level. While higher than expected returns resulting from cooperative harvesting in the past increase the importance of future resource productivity and foster sustainability, harvests below the reference level lead to a downward spiral of increasing overexploitation and disappointing returns.

## Introduction

Many social-ecological systems (SESs) that comprise a common pool resource (CPR) face the problem of overexploitation, because it is very costly, albeit not impossible, to exclude users from subtracting resource units [1]–[6]. Resource appropriation in such SESs often produces benefits for the individual, while all share the costs. This gives users an obvious incentive to maximize their harvest, thus, preventing cooperation and sustainability [7]. There is, however, compelling evidence both from economic experiments [8]–[11] and from real systems [12]–[15] that under certain conditions users may overcome the egoistic temptation of maximizing individual profits.

The decision to forgo part of a possible harvest from a renewable CPR is, particularly at low resource levels, an investment into future productivity at the cost of reduced short-term returns [16]. Because such an investment into the future always comes with uncertainties and because of the human preference for proximate returns, users discount potential future pay-offs [11], [17], [18]. According to the standard discounted utility model, rational users integrate all current and expected future returns after discounting them at a constant rate [17]. If discounted future benefits are large enough, users are willing to forego current benefits and cooperate [8], [10], [19].

Users of CPRs face highly uncertain decisions for two reasons. First, many ecological systems are characterized by high intrinsic variability, which complicates predictability [20]. Second, harvesting itself affects the stock and eventually the productivity of the resource. By this means, the harvest behavior may also influence the weight users assign to future resource productivity giving rise to a trade-off between the harvest behavior and the expected future returns [21]. The key to sustainability in many real SES is therefore to enhance the certainty of receiving the future benefits of cooperation, a goal achieved best by creating an institutional environment that is capable of accounting for the specific characteristics of the system under consideration [21]–[23]. Change, whether social or ecological, may, however, overstrain also robust institutions, when it is too rapid for successful adaptation [24].

Studies of real resource-user systems often disregard these close links between harvest behavior, resource dynamics, and future certainty and focus on either ecological or social aspects. The two sub-systems are described on different levels of detail, which hinders an integrated understanding of their coupled dynamics [25], [26]. Moreover, observational or experimental data from CPR systems usually cover only a short period of time or comprise many confounding factors and hence do not allow observing and understanding temporal changes. The quantitative relationship and the feedbacks between resource productivity and user behavior remain largely unknown. Consequently, regime shifts or collapses observed in overexploited ecological resources are still not fully understood [27], [28].

A novel approach for understanding the combined dynamics of a CPR and the users is to reduce the number of confounding factors by studying the system under controlled laboratory conditions. In a recent study, Janssen et al. [9] presented such a computer-based laboratory experiment, in which a group of five users could harvest continuously from a renewable CPR. Each group played six consecutive rounds of 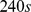 with a change of the treatment after the third round. In three of the six rounds neither communication nor punishment were possible (a treatment labeled as NCP), while in the other three rounds users could coordinate resource extraction using either communication (C), punishment (P), or a combination of both (CP).

Although the composition of each group was fixed and all properties of the game except the treatments were identical, the experimental results revealed a great variability of harvest behaviors within rounds, between rounds with the same treatment, as well as between different treatments. Users realized highest total harvests (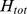) when they cooperated and allowed the CPR to grow and to produce more resource units at the beginning of a round. Communication, punishment (albeit to a lesser extend), and the total harvest realized in previous rounds influenced the harvest behavior of the users and their returns [9].

We combine here Janssen et al.’s experimental data with an adaptive model to identify the main drivers of the co-evolution of the users’ harvest behavior and the CPR. Our model is based on a mechanistic trade-off between the current harvest and the discount factor of future productivity. The trade-off accounts for the effect of resource exploitation on the certainty of future returns and reflects the central decision users face while harvesting from a renewable resource with a density-dependent growth. While maximizing the current harvest reduces the certainty of future returns, because the resource stock may decline significantly or even collapse as a consequence of intensive exploitation, lower current harvests enhance the chances of higher productivities and hence higher returns in the future.

Our model simulates a renewable CPR (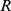, Equation 1) with a fixed number of users (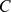), who realize a harvest (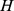, Equation 9) by adopting a variable harvest strategy. We define the harvest strategy as a continuous behavioral trait (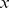, Equation 5) that determines the harvest rate of the users. 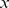 adapts to changes in 1) the current harvest opportunity and 2) the discounted future productivity, which we consider to be equivalent to the potential future returns. The CPR grows at a density-dependent logistic growth rate (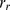, Equation 2) and users subtract variable amounts of resource units according to a Monod-type harvest rate (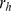, Equation 3). Changes in 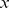 alter 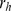 via the half-saturation constant (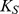, Equation 4), but also affect the discounted future productivity of the resource (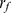, Equation 7).

More precisely, harvesting becomes less intense with increasing 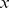, while the discount factor for future productivity (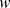, Equation 8) rises. The discount factor 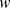, which is a function of the time horizon and the harvest trait, can vary between 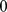 and the maximum discount factor (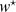). The parameter 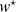 sets an upper limit for the weight of future productivity and represents the maximum level of certainty that is sustained by the social system, i.e. by the rules of the game, the institutions, and the experiences of the users. It is constant on short time-scales, because rules and institutions usually change slower than the fastest processes in ecological or social systems [24], but may vary between simulations accounting for different institutional environments or different harvest experiences.

Users maximize the net present value, which is the sum of the current pay-off and all discounted future pay-offs for given maximum discount factor and CPR level. Following adaptation models of continuous traits [29]–[32], the temporal change of the harvest trait 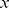 is proportional to the gradient of the fitness function 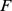, which is the sum of current and discounted future pay-offs (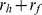) and the costs for optional punishment (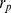, Equation 6). By adjusting 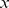, users change their harvest behavior to increase their fitness.

In behavioral economic experiments, user behavior is measured as cooperation. Cooperation is typically expressed as a dimensionless number between 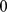 and 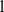 and determined by the user’s investment relative to a potential maximum value. Following this approach, we define the average cooperation of the group (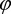) by the normalized foregone harvest (Equation 10), which is the amount of resource units that the users decide not to harvest divided by the maximum possible harvest.

In our model, users are not resolved as indidivuals. By contrast, the group of users is considered as a single adaptive entity, and the state variables of the model describe the dynamics of average group properties. The model hence corresponds to the typical resolution of observational data from real SESs and does not require detailed assumptions on the behavior of each individual in the system.

While Janssen et al. [9] focused on the statistical analysis of outcomes in terms of total harvest, our aim is to find a mechanistic explanation for the dynamics of the coupled system and the observed differences between rounds. Our major assumption is that the observed variability in harvest behavior and cooperation of users is caused by a trade-off between the current harvest and the discounted future productivity, which is mainly driven by differing maximum discount factors between rounds. Therefore, we 1) constrain the proposed trade-off by experimental data [9], 2) study the influence of the social environment, which is represented by a single parameter (the maximum discount factor 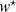), on the co-evolution of the user-CPR system, and 3) assess the effect of previous experience on the users’ perception of future certainty.

## Results and Discussion

### Trade-off between Harvest Behavior and Future Expectations

The expected future pay-offs of the CPR users were not measured directly during the experiments of Janssen et al. [9]. We instead use the relative resource productivity 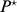 as a qualitative indication of the group’s expectations to constrain our model. 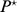 is defined as the cumulated resource productivity from the current time to the end of the experiment normalized to the resource level at the beginning of each round. In other words, 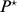 expresses the future productivity as a fraction of the initial resource level. Correspondingly, the inverse of the half-saturation constant 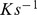, calculated from the experimental time-series of the harvest rate, is proportional to the resource affinity and used here equivalently. A high affinity value (or a low 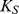) indicates aggressive harvesting already at low resource levels, while at low resource affinities users reach near maximum harvest rates only at high resource levels.

**Figure 1:**
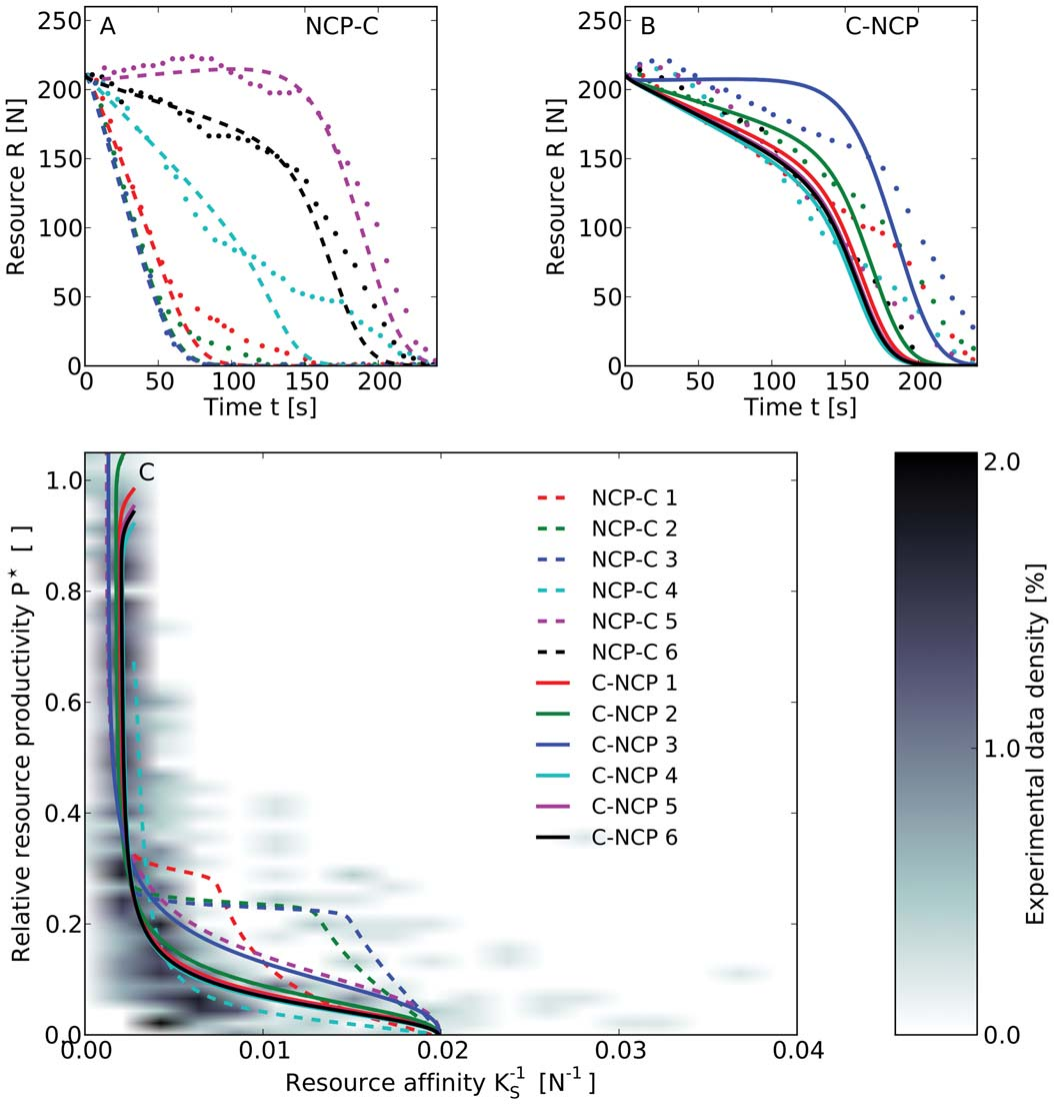
Trade-off between consumer’s resource affinity and the productivity of a renewable resource.

We discover a strong trade-off between 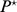 and 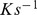 and a high variability of these two variables in the experimental data (Figure 1C). More specifically, in the experiment, relative resource productivities significantly exceeding 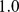 only occur at low resource affinities (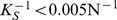). In contrast, high resource affinities (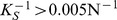) lead to large current harvests, while limiting the production of new resource units to values of 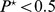. Similar to known relationships in real SESs (cf. Figure 2 in [26]), this trade-off is highly non-linear and introduces a tipping point to the system that clearly separates the effects of sustainable use from overexploitation. Users, thus, face the decision of increasing either short-term benefits or the long-term resource productivity [33].

**Figure 2:**
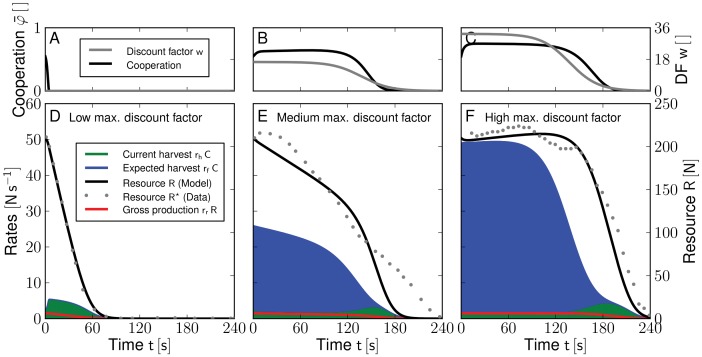
Time evolution of cooperation, harvest rates and a renewable resource for three different levels of future certainty. Increasing the maximum discount factor 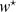 (Equation 8) lowers the current harvest 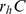 (Equation 3, lower panels **d**–**f**, green shaded area), but raises the future resource productivity 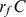 that is considered by the users (Equation 7, blue shaded area). Cooperation 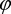 decreases sharply when future pay-offs are ignored (**a**, 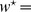
0.0) causing an immediate resource collapse (**d**, black solid line (model) and gray dots (experimental data from [9])). Larger values of 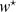 (**b**, 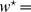
16.5 and **c**, 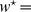
32.5) result in higher cooperation and reduce the current harvest as resource users account for a much higher proportion of future productivity (**e** and **f**). Resource collapse occurs later and the extended period of sustainable resource use leads to significantly higher total harvests (cf. Figures. S3a and S1). Red lines in panels **a**–**c** indicate the temporal evolution of the discount factor 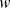.

**A–B**, Resource level in six rounds of a computer-based laboratory game with five users and different treatments (dotted lines indicate experimental data [9]). While in the first three rounds of **A** neither communication nor punishment was possible (NCP-C 1–3), users could communicate in subsequent rounds (NCP-C 4–6). In the treatment C-NCP (**A**), three rounds with communication (C-NCP 1–3) were followed by three NCP rounds (C-NCP 4–6). Set-ups of model runs (dashed and solid lines in **A** and **B**, respectively) only differ in the the maximum discount factor 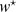 (see Equation 8, NCP-C: [4.4, 1.9, 1.4, 11.7, 32.5, 20.4], C-NCP: [18.0, 20.4, 29.3, 16.5, 17.3, 17.0] ). **C**, Phase plot of the users’ resource affinity, here defined as the inverse of the half-saturation constant 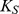 (see Equations 3 and 4), and the relative resource productivity 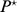, defined as the resource productivity from the current point of time to the end of a round normalized to the initial resource level. The shaded area shows the density distribution of the experimental data from the two treatments shown in **A** and **B**. Solid lines indicate the trade-off between resource affinity and potential future harvest from the resource system in corresponding model results.We calibrated our model to match the distribution of the experimental data and adjusted only the parameter 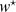 between rounds (Figure 1A and B). The model trajectories reveal the continuous change of user behavior over the course of the different rounds (Figure 1C). Starting from low affinities all model simulations end with 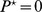 and 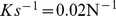, which represents the highest possible harvest rate and the complete exhaustion of the resource at the end of all rounds. While all rounds end similarly, they differ in the trajectories that lead to the exhaustion of the resource. When communication is possible (NCP-C 4–6, C-NCP 1–3), maximum 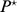 values are considerably higher than in NCP-rounds with no prior experience of communication (NCP-C 1–3). In contrast, users increase the resource affinity in NCP-C 1–3 right from the start (cf. Figure 1A and C) and by doing so avert high resource productivities.

### Effect of Different 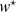 on the Temporal Dynamics of the User-CPR System

The different values of the maximum discount factor 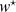 can be attributed to the changes in the social environment of the users, because the simulated rounds differ only by the available treatments and the history of previous round, whereas the resource characteristics and the composition of the groups of users were identical.

**Figure 3:**
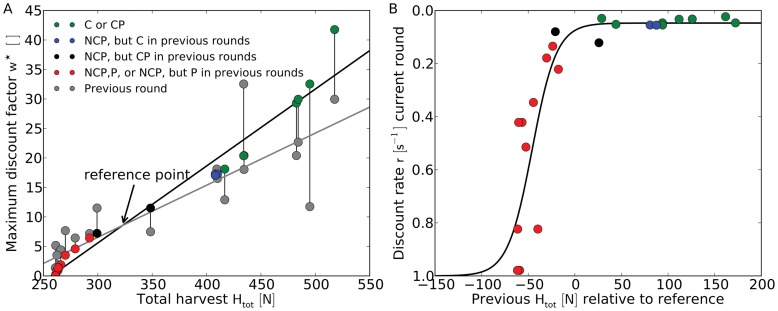
Outcomes of the common pool resource system. The total harvest 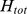 is closely related to the certainty of the future, here expressed as the maximum discount factor 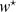 (Equation 8, colored dots). Only data from rounds that were preceded by a round with identical treatment (rounds 2, 3, 5, and 6) were included. **a**, Communication with or without punishment (CP and C, green dots) is essential to establish high 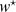 and to increase the 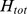. Punishment (P), even if experienced only in previous rounds, or the lack of communication and punishment (NCP), keep 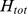 below the reference point (red dots). Blue and black dots indicate NCP rounds in which either communication or communication and punishment were available in previous rounds. The lines connecting two dots show the change of 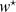 between a current and a preceding round (gray dots) with the same treatment (at the current round’s 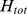). The intersection of the regression lines of previous (gray line) and current (black line) discount factors reveals that the value of 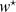 increases from round to round if the group manages to establish a 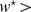
8.5 (corresponding to 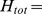
323) in the preceding round, or decreases if 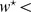
8.5. This intersection marks the sustainability threshold between positive and negative feedbacks in the system and sets the reference point for the users’ expectations. **b**, 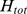 in a preceding round determines the discount rate 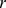, derived from the relation 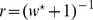. The solid, sigmoidal line indicates a least-squares fit to a logistic equation (root mean square error 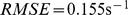).

If the future is irrelevant to users and future returns are disregarded (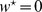, Figure 2A and D), cooperation levels deteriorate within the first 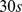 of the simulation. In this case, the current harvest rate significantly exceeds the growth rate of the CPR and the unsustainable use leads to a collapse within the first 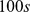 and to a poor total harvest (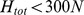, Figure 3A). Increasing the importance of the future, that is increasing 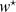, results in higher cooperation over longer periods of time and slows down (Figure 2B and E) or even reverses overexploitation (Figure 2C and F). Towards the end of all simulations, however, 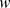 declines with the remaining time of the experiment causing an erosion of cooperation that eventually triggers the collapse of the resource (Figure 2A–C), because there is no potential future productivity to account for in a finite game.

Users continuously adjust their harvest strategy according to changing present harvest opportunities and expected future pay-offs. Therefore, cooperation and sustainable harvesting become rational when the discounted total harvest for one strategy is higher than for other strategies [34]. By treating resource users as an adaptive entity, our model unveils their great behavioral variability and the smooth transition from a sustainable to unsustainable resource use. These results support studies [35] that question stable norms of cooperation derived from “one-shot” field experiments [36]–[39]. The observed behavioral variability among CPR users with identical cultural background corroborates our assumption that users adapt their harvest behavior to the properties of the social and ecological environment [13], [35], [40].

### Relation between the Maximum Discount Factor and the Total Harvest

The harvest behavior of CPR users and hence the outcome of the artificial commons vary greatly between rounds (cf. Figure S1 and S2 for the results of all rounds). A quantitative measure that may explain those differences of the total harvest 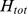 is the maximum discount factor 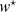. The total harvest indicates the success of the group’s behavior and is positively related to 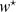 (Figure 3A), because in the model the productivity increases with 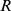 for the range of resource levels observed in the experiment (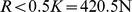). In other words, a high 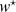 leads to harvest rates below productivity, i.e. sustainability, at the beginning of a round and eventually to high total harvests. Given the constraints of the experiment, the highest 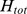 of 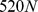 is therefore realized at the highest value of 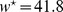 (Figure 3A).

By only adjusting 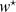 to match the experimental results, we assume that differences between rounds are mainly caused by variations in the users’ perception of future certainty. Allowing for variations also in the parameters 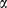 and 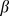 reduces the error between model and experimental data indicating that users also adjust the temporal dynamics of the harvest from round to round (Figure S4 and Table S1). However, the results of a systematic sensitivity analysis (Figure S5) confirm the high sensitivity of the model results to changes in 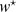 and corroborate the choice of 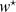 as the only free parameter explaining the observed differences between the rounds of the experiment.

### Effect of Different Treatments on the Maximum Discount Factor

Our analysis reveals that the variations between rounds in the perception of future certainty, represented by 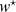 in the model, are determined by a combination of factors, including 1) treatment in the current round, 2) prior experience from rounds with the same treatment, and 3) possible exposure to a different treatment in the past.

Sustainable harvest strategies, characterized by high 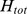 exceeding 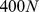, are associated with rounds in which either communication is possible or users had experienced communication in previous rounds (Figure 3A). On the contrary, in the absence of communication or with prior experience of punishment, users harvest unsustainably throughout the simulation and, thus, realize poor harvests (below 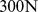). Rounds in which the positive and negative effects of previous communication and punishment are balanced represent a transition between clearly separated strategies of successful and unsuccessful harvests (black dots in Figure 3A).

The impact of previous experience of punishment on the maximum discount factor and therefore on the total harvest becomes clear when comparing the outcomes of NCP rounds that were preceded by differing treatments. While in rounds 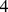 and 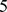 of the C-NCP treatment users manage to sustain a 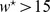, the maximum discount factor drops considerably to values around 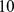 in corresponding rounds of the CP-NCP treatment (Figure 3A). In our model, this indirect effect on the harvest behavior is much stronger than any direct effect of punishment. As a tool to enhance the confidence of users into the future, punishment has, different from communication, no or even negative effects beyond the period of its availability [41]. Punishment alters the expectations of future returns, but communication is clearly more effective in raising the users’ maximum discount factor and eventually in establishing cooperation [9], [42], [43]. In studies of real social-ecological systems, leadership significantly influences the successful management of the commons [12], [13], [44]. Supported by Janssen et al. ’s observations [9], we argue that communication enables negotiation and promotes leadership in the artificial environment of this simple, computer-based CPR system.

### Changes of the Maximum Discount Factor within One Treatment

A feedback between 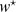 and 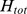 links the outcome of previous rounds with the same treatment to the current harvest strategy. Our results suggest that the group’s total harvest will increase further in the following round with identical treatment if 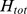 is above a certain threshold (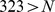, equivalent to 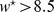). Values below this reference lead to a further deterioration of the maximum discount factor and diminish the group’s total harvest in most rounds.

The reference value hence marks a sustainability threshold for the system. This feedback between rounds is similar to the mechanism proposed by Fehr & Gächter [45], who explained the decay of cooperation in a public goods game as a feedback loop of disappointed expectations that leads to lower and lower endowments of the players of the experiment.

The discount rates 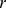, which were derived from 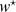 assuming exponential discounting [17], are related to the 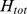 of the previous round by a sigmoid function (Figure 3B). We suggest that the threshold determined in Figure 3A and the discount rate correspond respectively to the reference point of the value function and to the psychological value of an outcome in prospect theory [46]. Prospect theory, which is based on gains and losses rather than on absolute outcomes, explains the discrepancies between economic rationality and observed human behavior. According to prospect theory the perceived value of an outcome does not depend linearly on its economic value. Instead, it is an asymmetric, sigmoidal function of gains and losses with respect to a reference point, the value function, which can be influenced by expectations or the current status. Furthermore, the weight humans associate to an uncertain outcome is related, but not equal, to the corresponding probability, because the human ability to objectively estimate probabilities, in particular those of rare events, is limited [46], [47].

We, thus, argue that users adjust the discount rates according to the psychological value of the total harvest realized in the previous round with identical treatment. Users are highly sensitive to losses, which are caused in this system by the lack of communication or by punishment. Losses with respect to the reference value in the previous round lead to a severely impaired perception of future certainty. In contrast, small gains can be sufficient for users to adopt lower discount rates in following rounds (Figure 3B). Note, however, that we excluded the first rounds with a new treatment from our analysis, because a drastic change of the institutional environment does clearly affect future expectations and obfuscates the relationship between harvest experience and discounting as shown in Figures 3A and B.

### Conclusions

We have shown that it is possible to understand main features of a CPR and the harvest dynamics of a simplified SES by reducing the social environment to its impact on the perceived future certainty of the users. Our approach extends classic models of maximum sustainable [48], [49] or maximum economic [34] yield by introducing a behavioral trait that accounts for the mutual dependency of current behavior and future expectations. The social environment including the first-tier variables “users” and “governance system” of Ostrom’s framework for the analysis of SESs [23] is obviously more complex than assumed here and exhibits a dynamics of its own (indicated in Figure 3A and 3B). Despite this well-recognized complexity, the harvest behavior of CPR users can be analyzed, understood, and even roughly predicted with a simple model. Our model is able to describe the co-evolution of the renewable CPR and the adaptive harvest behavior of the users following a mechanistic trade-off, a disregarded feature in classic harvest models [16]. Furthermore, by showing the influence of user experience on the perception of future certainty, we presented an approach to understand the observed variability of user behavior in apparently similar or identical situations.

We conclude that unsustainable harvest leads to reduced discounted future pay-offs and low cooperation in two ways, first, as a consequence of reduced resource productivity and, second, as a consequence of a deteriorating discount factor. Once the temporal gradient of both terms has turned negative, it is difficult for users to escape from the downward vortex of decreasing expectations and diminishing pay-offs. This feedback works also in the opposite direction towards sustainable harvest strategies, high pay-offs, and sustained cooperation among resource users. Our findings illustrate the behavioral variability of users that act rationally according to their current opportunities and their perception of future returns. By this means, our approach opens up a perspective for predicting dynamics and identifying tipping points of coupled user-resource systems.

## Methods

Our adaptive model consists of three ordinary differential equations and describes the combined dynamics of the resource 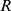, the harvest 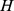, and the harvest trait 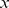 for a constant number of users 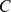.

### The Renewable Resource


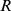
 is changing over time 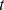 according to the difference between new production and harvest
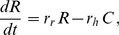
(1)where 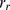 is the productivity of 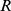


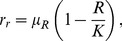
(2)with 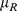 and 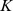 indicating the maximum specific growth rate and the carrying capacity of the resource system, respectively. Resource growth is hence logistic with highest growth rates at 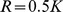 and declining rates towards 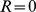 as well as towards 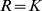 [16].

### Adaptive Harvesting

The current harvest rate 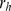 is based upon Monod kinetics
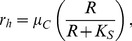
(3)with 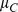 representing the maximum specific harvest rate and 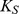 the half-saturation constant. The harvest rate 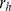 is, thus, insensitive to changes in 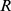 for 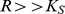, but sensitive for 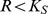. In our model, 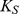 is variable and responds to changes in the harvest trait 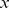.
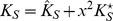
(4)where 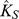 and 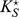 denote the minimum and the variable part of 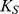, respectively. Using an adaptive modeling approach [29]–[32], the temporal change of 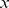 is proportional to the fitness gradient 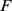


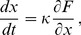
(5)with 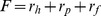 and 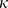 denoting the rate constant of the adaptive process [29]. 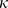 hence parameterizes the speed of the adaption process, i.e. the speed of learning, of the group of users over the course of a round. While the punishment rate
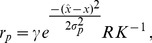
(6)is only available in some rounds, the discounted future productivity
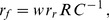
(7)which stands for the future resource productivity expected by the users, is considered in all rounds. In Equation 6 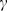 is the specific punishment rate, 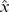 is the trait value of the punishment maximum, and 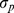 is the punishment standard deviation. Consistent with the experimental results [9], 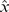 is set to intermediate values in the model so that punishment is applied mostly at intermediate levels of 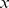. At high 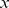 users forgo a large fraction of their potential harvest, they cooperate, and punishment is therefore not necessary. By contrast, at low levels of 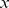, which indicate egoistic harvest strategies and low importance of future pay-offs, users are not inclined to invest in a costly and uncertain measure that may support long-term sustainability. The discount factor 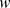 is a variable function of 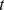 and 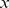


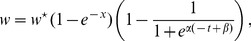
(8)with 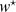, the maximum discount factor, representing the only free parameter between rounds, and 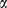 and 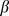, two shape parameters, determining the decay of 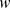 as the time in a round elapses. While the time dependence of 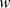 is similar to the discounted utility model [17], the parameters 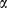 and 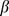 allow for a modification of the timing and the speed of decay of 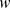 with time. 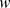 acts here as a weight on future productivities and is connected to the harvest behavior of the users via the harvest trait 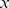. We assume here that users estimate the future productivity of the resource at the current resource level. Hence, the trade-off between 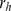 and 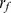 emerges, because an increase of 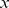 raises the current harvest 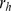, but erodes 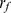 by reducing 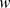. The functional dependence of 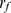 and 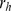 on 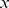 is determined by the highly non-linear shape of the trade-off in the data (cf. Figure 1C) and constrained by the requirement that 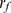 and 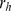 may not be negative for any 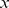. Integrating the harvest over time while accounting for possible costs for punishment gives the temporal evolution of the harvest 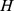




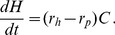
(9)The total harvest realized over the 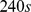 of an experimental round is then 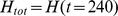.

### Cooperation

Cooperation is a diagnostic variable in our model. It is defined by the non-realized current harvest normalized to the maximum possible harvest 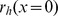


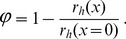
(10)


In other words, not harvesting anything results in 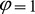, whereas maximizing the current harvest rate by adopting 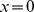 leads to 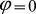. Note that considering users as an adaptive entity implies that the properties 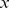 and also 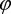 are mean properties of the group. Unlike in similar evolutionary dynamics models, the dynamics of the trait distribution is not determined by the reproductive fitness of individuals bearing a certain trait, because we assume that the change of a behavioral trait does not require sexual reproduction [50]. In our model, the fixed group of resource users is able to quickly adapt the harvest strategy according to the state of the resource and the maximum discount factor, an assumption that is corroborated by the observed variability of harvest rates in the laboratory experiments [9]. Consistent with the published results of the experimental study, the model is not spatially explicit, because the dynamics of the spatial averages of the resource and the group’s harvest in the homogeneous system can be adequately described by zero-dimensional approach.

### Simulations

The parameter-set of the model was manually calibrated to fit the temporal evolution of the resource and the total harvest observed in the 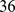 rounds of the laboratory experiment (cf. Table 1 and Figure S2). The data [9] are averages of five or six replicates for each round. All simulations were conducted with identical initial conditions and parameter values except for the maximum discount factor 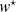. Changes of 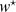 account for all variability in the model results we show in the main text. The different treatments and the learning of the users are, thus, reduced to their impact on the expectations of future pay-off, which is represented by the maximum discount factor 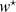 in the model. Additional results with three variable parameters are presented in the Supporting Information (Figures S4 and S5, Text S1, and Table S1).

**Table 1:** Parameter values and variables (with initial conditions given in parenthesis).

Symbol	Name	Value	Unit
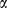	Shape parameter	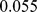	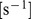
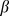	Shape parameter	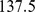	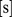
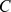	Users	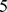	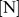
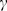	Specific punishment rate	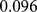	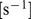
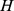	Harvest	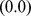	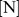
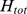	Total harvest	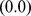	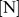
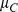	Max. specific harvest rate	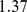	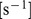
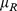	Max. specific resource growth rate	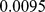	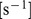
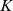	Carrying capacity	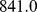	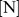
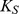	Half-saturation const.	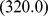	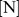
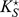	Variable half-saturation const.	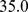	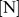
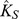	Min. half-saturation const.	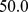	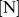
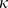	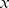 Variance of	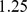	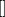
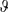	Cooperation	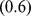	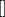
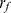	Future productivity	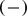	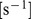
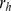	Current harvest rate	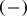	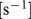
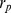	Punishment rate	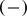	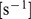
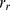	Resource growth rate	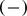	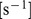
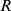	Resource	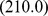	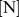
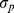	Punishment standard deviation	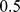	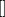
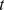	Time	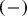	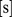
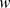	Discount factor	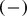	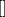
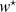	Max. discount factor	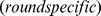	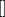
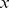	Mean harvest trait	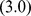	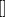

## Supporting Information

Figure S1
**Total harvest for different treatments.** Comparison of total harvest 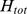 for combinations of different treatments, namely communication (C), costly punishment (P), communication and costly punishment (CP), neither communication nor punishment (NCP). Respectively, bars and dots with error bars denote mean values and standard deviations of experimental results obtained from the laboratory study of Janssen et al. [9].(TIF)

Figure S2
**Temporal dynamics of the resource.** Times-series of resource levels for six experiments consisting of six rounds each. Treatments are communication (C), costly punishment (P), communication and costly punishment (CP), neither communication nor punishment (NCP) and change after three rounds. Solid lines indicate model results and dotted lines indicate the experimental results [9]. Only the maximum discount factor 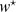 was varied between rounds to fit the experimental results, all other parameter values are reported in Table 1.(TIF)

Figure S3
**Model-data comparison.** Comparison of all experimental data from [9] shown in Figure S2 with corresponding model data. A linear regression yields 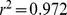.(TIF)

Figure S4
**Temporal dynamics of the resource.** Times-series of resource levels for six experiments consisting of six rounds each. Treatments are communication (C), costly punishment (P), communication and costly punishment (CP), neither communication nor punishment (NCP) and change after three rounds. Solid lines indicate model results and dotted lines indicate the experimental results obtained from the laboratory study of Janssen et al. [9]. Only the parameters 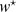, 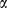, and 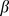 vary between rounds to fit the experimental results, all other parameter values are reported in Table 1.(TIF)

Figure S5
**Sensitivity analysis.** Sensitivity of the root mean square (RMS) error between simulated and experimental data to changes in the three parameters 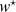 (A), 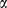 (B), and 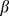 (C), all other parameter values are reported in Table 1. The ranges of variation were 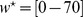, 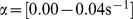, and 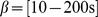. Each of the 36 experimental resource time-series was compared to the results of 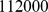 model runs with unique combinations of the three variable parameters to find the optimal parameter values (cf. Figure S4 for the best results). The panels A–C show how the RMS error increases from the optimum when only one of the three parameters is varied while the other two are held constant at their optimum value.(TIF)

Table S1
**Root mean square error (RMSE) between experimental and simulated data for models with one (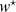) and three free parameters (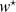, 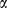, and 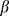).**
(PDF)

Text S1 **Additional model results.**
(PDF)

## References

[1] Pauly D, Christensen V, Dalsgaard J, Froese R, Torres Jr F (1998) Fishing down marine food webs. Science 279: 860–863. 9452385 10.1126/science.279.5352.860

[2] Pauly D, Christensen V, Guenette S, Pitcher TJ, Sumaila UR, et al (2002) Towards sustainability in world fisheries. Nature 418: 689–695. 12167876 10.1038/nature01017

[3] Jackson JB, Kirby MX, Berger WH, Bjorndal KA, Botsford LW, et al (2001) Historical overfishing and the recent collapse of coastal ecosystems. Science 293: 629–37. 11474098 10.1126/science.1059199

[4] Achard F, Eva H, Stibig H, Mayaux P, Gallego J (2002) Determination of deforestation rates of the world’s humid tropical forests. Science 297: 999–1002. 12169731 10.1126/science.1070656

[5] Pandolfi J, Jackson J, Baron N, Bradbury R (2005) Are US coral reefs on the slippery slope to slime? Science 307: 1725–1726. 15774744 10.1126/science.1104258

[6] Wada Y, van Beek LPH, van Kempen CM, Reckman JWTM, Vasak S, et al (2010) Global depletion of groundwater resources. Geophys Res Lett 37: 1–5.

[7] Levin SA (2010) Crossing scales, crossing disciplines: collective motion and collective action in the Global Commons. Philos Trans R Soc London, Ser B 365: 13–8. 20008381 10.1098/rstb.2009.0197 PMC2842704

[8] Dal Bó P, Fréchette G (2011) The evolution of cooperation in in_netely repeated games: experimental evidence. Amer Econ Rev 101: 411–429.

[9] Janssen MA, Holahan R, Lee A, Ostrom E (2010) Lab experiments for the study of social-ecological systems. Science 328: 613–617. 20431012 10.1126/science.1183532

[10] Dal Bó P (2005) Experimental cooperation under the shadow of the future: evidence from infitely repeated games. Amer Econ Rev 95: 1591–1604.

[11] Gintis H (2000) Beyond Homo economicus: evidence from experimental economics. Ecol Econ 35: 311–322.

[12] Gutiérrez NL, Hilborn R, Defeo O (2011) Leadership, social capital and incentives promote successful fieries. Nature 470: 386–9. 21209616 10.1038/nature09689

[13] Rustagi D, Engel S, Kosfeld M (2010) Conditional Cooperation and Costly Monitoring Explain Success in Forest Commons Management. Science 330: 961–965. 21071668 10.1126/science.1193649

[14] Dolsak N, Ostrom E, editors (2003) The Commons in the New Millennium: Challenges and Adaptations. Cambridge: The MIT Press.

[15] Ostrom E (1990) Governing the Commons. Cambridge: Cambridge University Press.

[16] Clark C (1976) Mathematical Bioeconomics. New York: Wiley.

[17] Frederick S, Loewenstein G, O’Donoghue T (2002) Time discounting and time preference: a critical review. J Econ Lit XL: 351–401.

[18] Holt Ca, Laury SK (2002) Risk aversion and incentive effects. Amer Econ Rev 92: 1644–1655.

[19] Axelrod R (1984) The Evolution of Cooperation. New York: Basic Books.

[20] Scheéér M, Carpenter S, Foley JA, Folke C, Walker B (2001) Catastrophic shifts in ecosystems. Nature 413: 591–6. 11595939 10.1038/35098000

[21] Kortenkamp KV, Moore CF (2006) Time, uncertainty, and individual differences in decisions to cooperate in resource dilemmas. Pers Soc Psychol B 32: 603–615. 10.1177/0146167205284006 16702154

[22] Ostrom E (2009) A general framework for analyzing sustainability of social-ecological systems. Science 325: 419–422. 19628857 10.1126/science.1172133

[23] Ostrom E (2007) A diagnostic approach for going beyond panaceas. Proc Nat Acad Sci USA 104: 15181–15187. 17881578 10.1073/pnas.0702288104 PMC2000497

[24] Dietz T, Ostrom E, Stern PC (2003) The struggle to govern the commons. Science 302: 1907–12. 14671286 10.1126/science.1091015

[25] An L (2011) Modeling human decisions in coupled human and natural systems: Review of agentbased models. Ecol Modell 229: 25–36.

[26] Liu J, Dietz T, Carpenter SR, Alberti M, Folke C, et al (2007) Complexity of coupled human and natural systems. Science 317: 1513–6. 17872436 10.1126/science.1144004

[27] Pinsky ML, Jensen OP, Ricard D, Palumbi SR (2011) Unexpected patterns of fisheries collapse in the world’s oceans. Proc Nat Acad Sci USA 108: 8317–8322. 21536889 10.1073/pnas.1015313108 PMC3100948

[28] Lees K, Pitois S, Scott C, Frid C, Mackinson S (2006) Characterizing regime shifts in the marine environment. Fish Fish 7: 104–127.

[29] Abrams Pa, Matsuda H, Harada Y (1993) Evolutionarily unstable fitness maxima and stable fitness minima of continuous traits. Evol Ecol 7: 465–487.

[30] Wirtz KW, Eckhardt B (1996) Effective variables in ecosystem models with an application to phytoplankton succession. Ecol Modell 92: 33–53.

[31] Norberg J, Swaney DP, Dushoff J, Lin J, Casagrandi R, et al (2001) Phenotypic diversity and ecosystem functioning in changing environments: a theoretical framework. Proc Nat Acad Sci USA 98: 11376–11381. 11535803 10.1073/pnas.171315998 PMC58737

[32] Merico A, Bruggeman J, Wirtz KW (2009) A trait-based approach for downscaling complexity in plankton ecosystem models. Ecol Modell 220: 3001–3010.

[33] Janssen MA, Anderies JM (2007) Robustness trade-offs in social-ecological systems. Int J Comm 1: 43–66.

[34] Grafton RQ, Kompas T, Hilborn RW (2007) Economics of overexploitation revisited. Science 318: 1601. 18063793 10.1126/science.1146017

[35] Lamba S, Mace R (2011) Demography and ecology drive variation in cooperation across human populations. Proc Nat Acad Sci USA 2011: 1–5. 10.1073/pnas.1105186108 PMC3167540 21831836

[36] Gächter S, Herrmann B (2009) Reciprocity, culture and human cooperation: previous insights and a new cross-cultural experiment. Philos Trans R Soc London, Ser B 364: 791–806. 19073476 10.1098/rstb.2008.0275 PMC2689715

[37] Herrmann B, Thöni C, Gächter S (2008) Antisocial punishment across societies. Science 319: 1362–1367. 18323447 10.1126/science.1153808

[38] Henrich J (2004) Cultural group selection, coevolutionary processes and large-scale cooperation. J Econ Behav Organ 53: 3–35.

[39] Henrich J, Boyd R, Bowles S, Camerer C, Fehr E, et al (2001) In search of Homo economicus: behavioral experiments in 15 small-scale societies. Amer Econ Rev 91: 73–78.

[40] Hayo B, Vollan B (2012) Group interaction, heterogeneity, rules, and co-operative behaviour: evidence from a common-pool resource experiment in South Africa and Namibia. J Econ Behav Organ 81: 9–28.

[41] Gneezy URI, Rustichini A, Glaeser E, Levine D, Nyhus E, et al (2000) A fine is a price. J Legal Stud XXIX: 1–17.

[42] Rand DG, Dreber A, Ellingsen T, Fudenberg D, Nowak MA (2009) Positive interactions promote public cooperation. Science 325: 1272–5. 19729661 10.1126/science.1177418 PMC2875121

[43] Dreber A, Rand DG, Fudenberg D, Nowak MA (2008) Winners don’t punish. Nature 452: 348–51. 18354481 10.1038/nature06723 PMC2292414

[44] Kenward RE, Whittingham MJ, Arampatzis S, Manos BD, Hahn T, et al (2011) Identifying governance strategies that effectively support ecosystem services, resource sustainability, and biodiversity. Proc Nat Acad Sci USA 108: 5308–12. 21402916 10.1073/pnas.1007933108 PMC3069166

[45] Fehr E, Fischbacher U (2003) The nature of human altruism. Nature 425: 785–791. 14574401 10.1038/nature02043

[46] Kahneman D, Tversky A (1979) Prospect theory: an analysis of decision under risk. Econometrica 47: 263–292.

[47] Tversky A, Kahneman D (1974) Judgment under uncertainty: heuristics and biases. Science 185: 1124–1131. 17835457 10.1126/science.185.4157.1124

[48] Lande R, Engen S, Saether B (1994) Optimal harvesting, economic discounting and extinction risk in uctuating populations. Nature 372: 88–90.

[49] Clark C (1973) The economics of overexploitation. Science 181: 630–634. 17736970 10.1126/science.181.4100.630

[50] Levin SA (2006) Learning to live in a global commons: socioeconomic challenges for a sustainable environment. Ecol Res 21: 328–333.

