# Supplementary figures and images for: Human Adaptive Behavior in Common Pool Resource Systems
Source: PLoS One. 2012 Dec 28;7(12):e52763. doi: 10.1371/journal.pone.0052763 (PMC3532302; doi:10.1371/journal.pone.0052763)

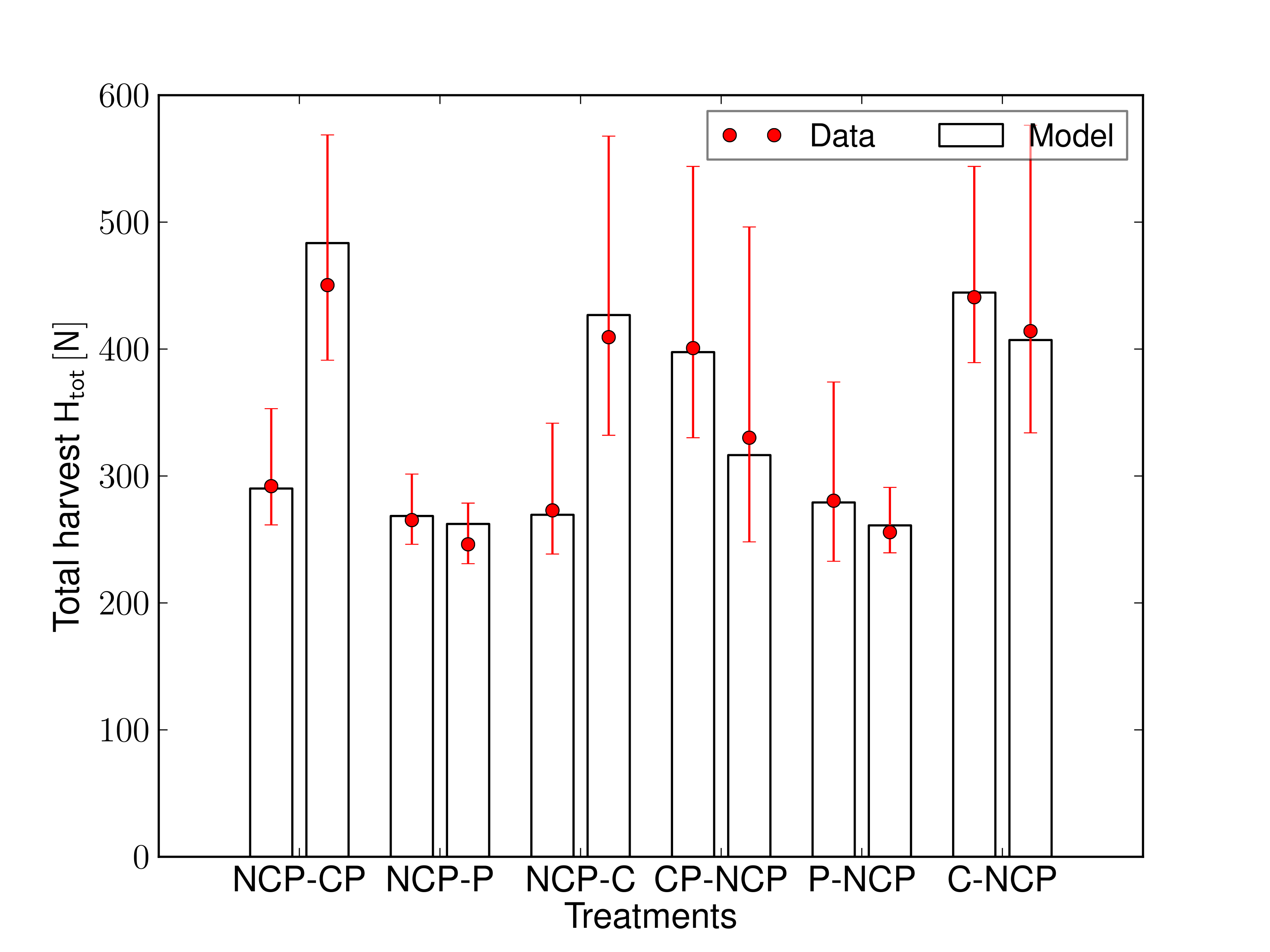

Supplement: Figure S1 — Total harvest for different treatments. Comparison of total harvest for combinations of different treatments, namely communication (C), costly punishment (P), communication and costly punishment (CP), neither communication nor punishment (NCP). Respectively, bars and dots with error bars denote mean values and standard deviations of experimental results obtained from the laboratory study of Janssen et al. [9]. (TIF) [file pone.0052763.s001.tif]

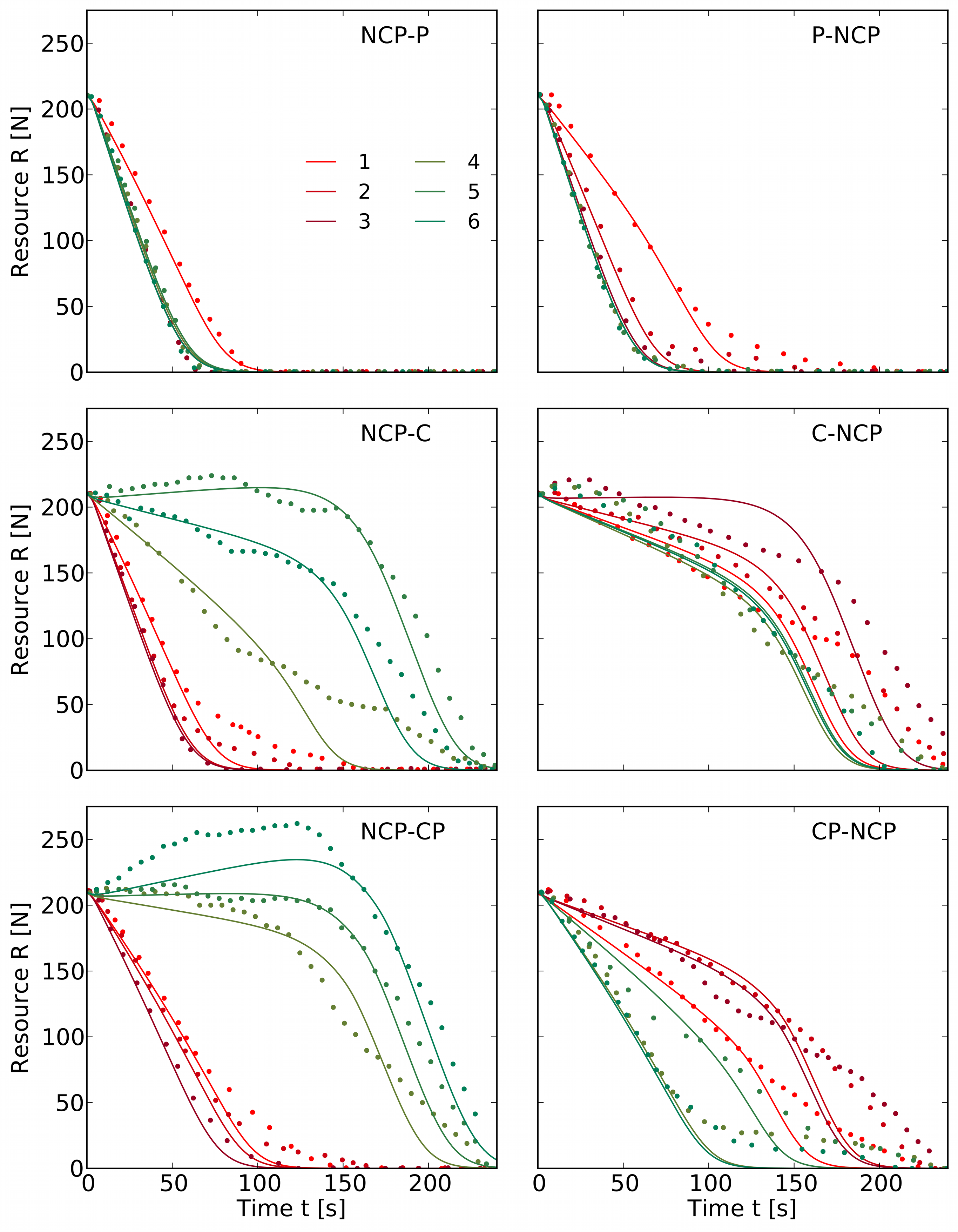

Supplement: Figure S2 — Temporal dynamics of the resource. Times-series of resource levels for six experiments consisting of six rounds each. Treatments are communication (C), costly punishment (P), communication and costly punishment (CP), neither communication nor punishment (NCP) and change after three rounds. Solid lines indicate model results and dotted lines indicate the experimental results [9]. Only the maximum discount factor was varied between rounds to fit the experimental results, all other parameter values are reported in Table 1. (TIF) [file pone.0052763.s002.tif]

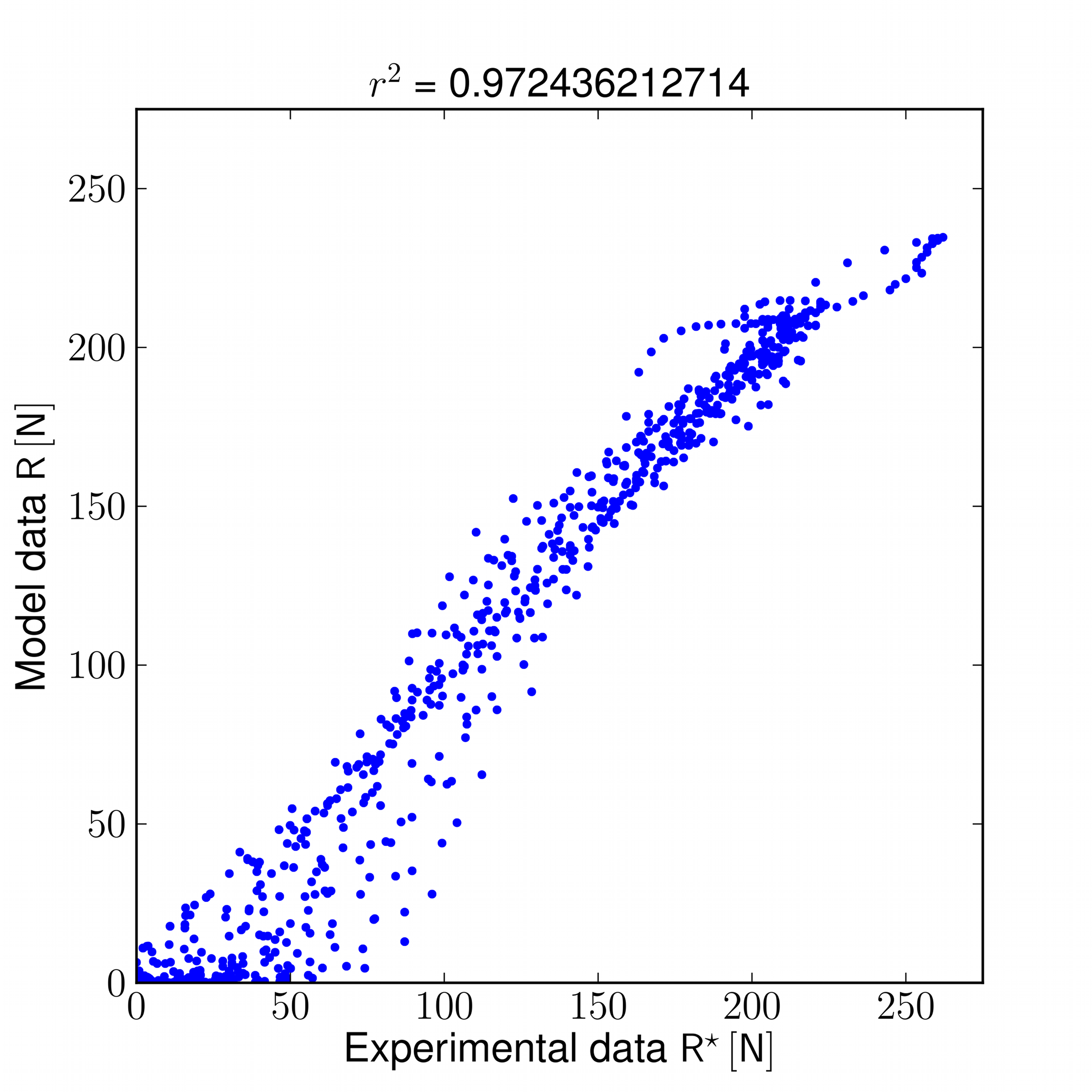

Supplement: Figure S3 — Model-data comparison. Comparison of all experimental data from [9] shown in Figure S2 with corresponding model data. A linear regression yields . (TIF) [file pone.0052763.s003.tif]

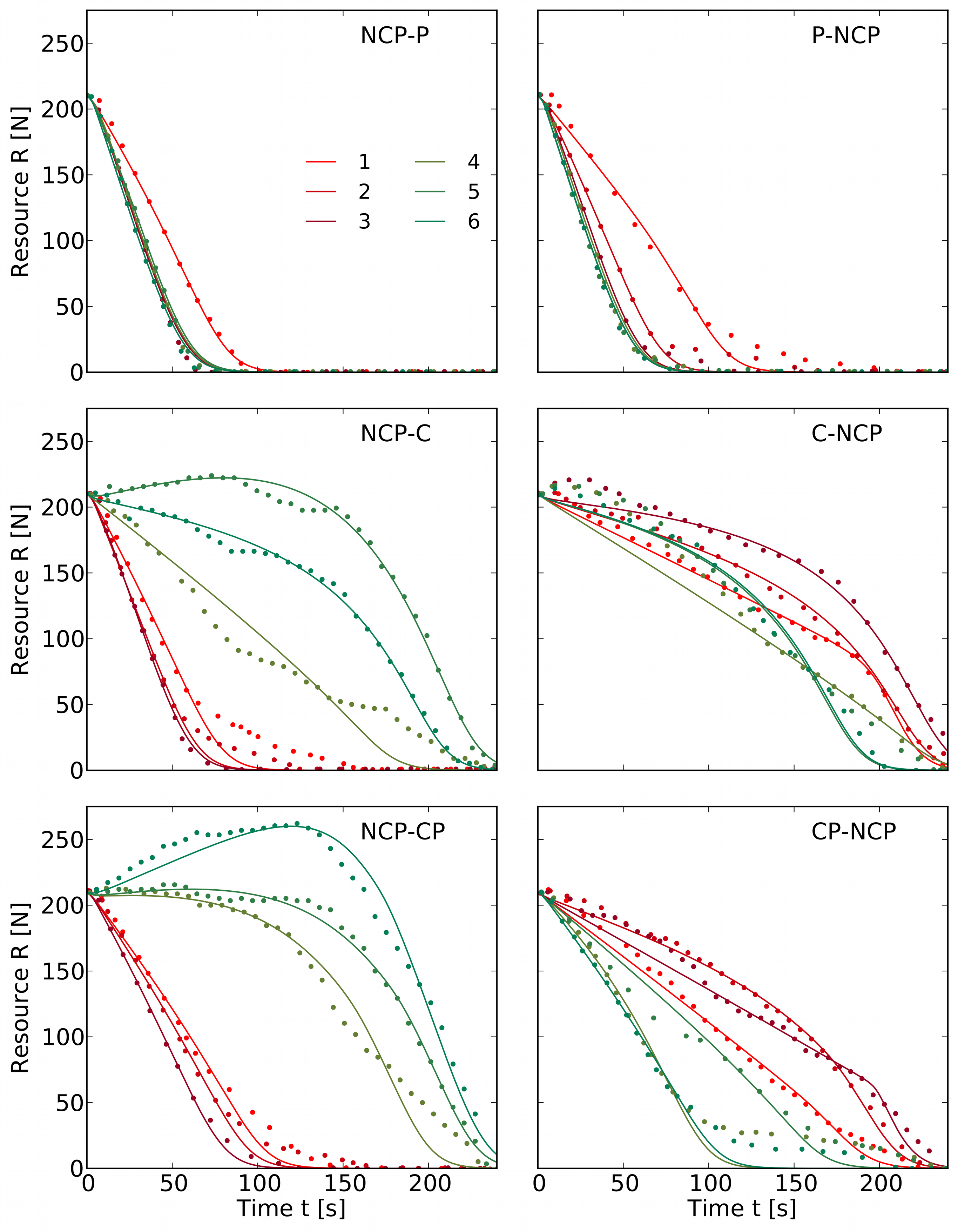

Supplement: Figure S4 — Temporal dynamics of the resource. Times-series of resource levels for six experiments consisting of six rounds each. Treatments are communication (C), costly punishment (P), communication and costly punishment (CP), neither communication nor punishment (NCP) and change after three rounds. Solid lines indicate model results and dotted lines indicate the experimental results obtained from the laboratory study of Janssen et al. [9]. Only the parameters , , and vary between rounds to fit the experimental results, all other parameter values are reported in Table 1. (TIF) [file pone.0052763.s004.tif]

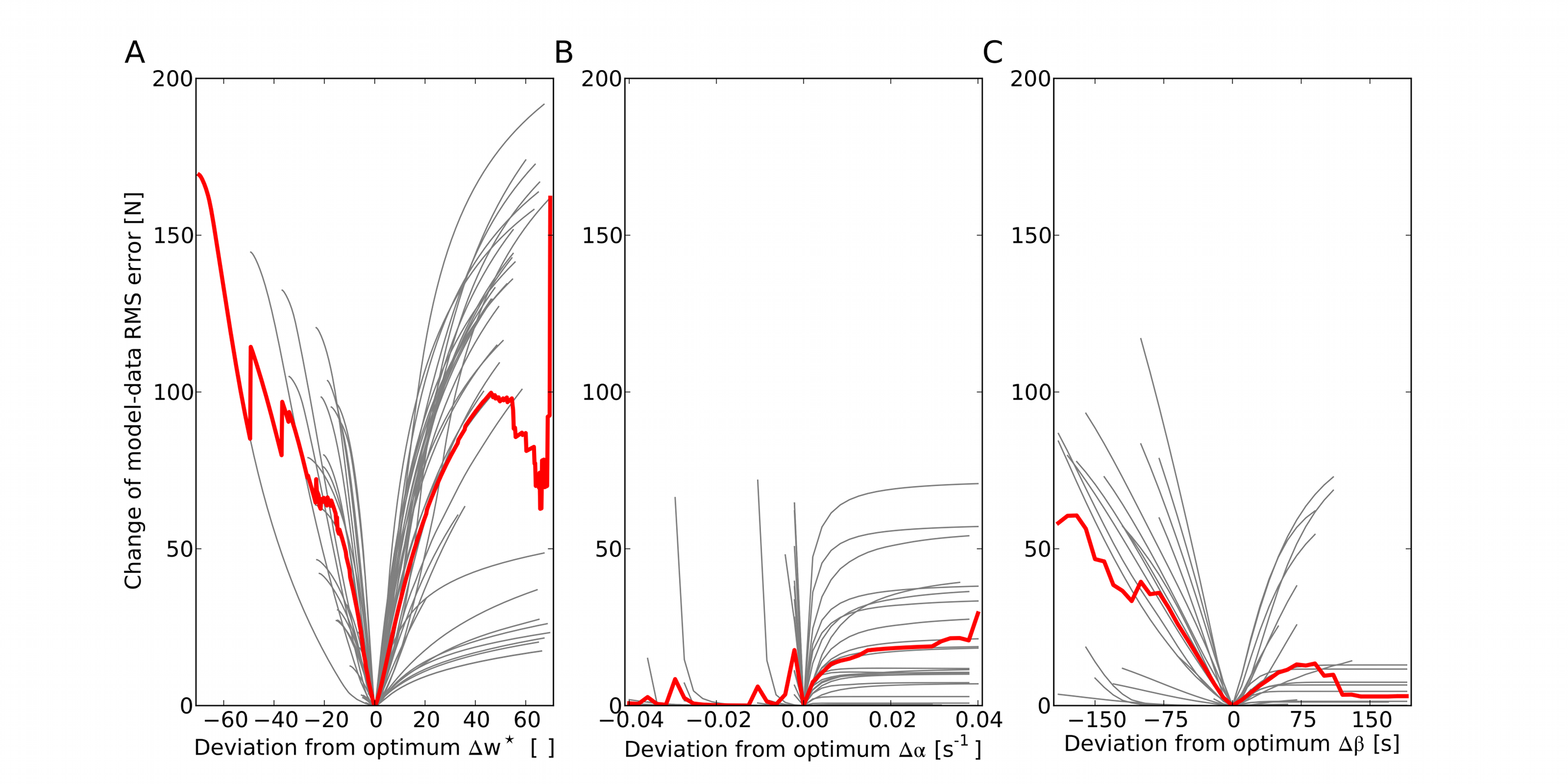

Supplement: Figure S5 — Sensitivity analysis. Sensitivity of the root mean square (RMS) error between simulated and experimental data to changes in the three parameters (A), (B), and (C), all other parameter values are reported in Table 1. The ranges of variation were , , and . Each of the 36 experimental resource time-series was compared to the results of model runs with unique combinations of the three variable parameters to find the optimal parameter values (cf. Figure S4 for the best results). The panels A–C show how the RMS error increases from the optimum when only one of the three parameters is varied while the other two are held constant at their optimum value. (TIF) [file pone.0052763.s005.tif]
