# Supplementary material for: Human Adaptive Behavior in Common Pool Resource Systems
Source: PLoS One. 2012 Dec 28;7(12):e52763. doi: 10.1371/journal.pone.0052763 (PMC3532302; doi:10.1371/journal.pone.0052763)
Supplement: Table S1 — Root mean square error (RMSE) between experimental and simulated data for models with one () and three free parameters (, , and ). (PDF) [file pone.0052763.s006.pdf]

Table S1. Root mean square error (RMSE) between experimental and simulated data for models with one ($w^\star$) and three free parameters ($w^\star$, α, and β).

Treatment	Round	$\text{RMSE}_{w^\star}$	$\text{RMSE}_{w^\star\alpha\beta}$
NCP-P	1	3.9	1.6
	2	3.2	2.3
	3	3.2	2.3
	4	2.9	1.4
	5	3.5	1.7
	6	2.9	2.5
P-NCP	1	7.2	6.3
	2	6.2	3.7
	3	3.9	2.0
	4	2.7	2.2
	5	2.3	2.3
	6	1.8	1.8
NCP-C	1	9.3	8.0
	2	6.4	4.5
	3	3.2	1.8
	4	18.3	12.9
	5	10.9	3.8
	6	10.9	3.1
C-NCP	1	20.3	2.9
	2	15.2	3.4
	3	24.3	5.5
	4	18.1	10.8
	5	13.7	10.3
	6	10.1	6.2
NCP-CP	1	6.5	4.5
	2	6.3	4.7
	3	3.1	1.6
	4	13.6	8.9
	5	8.5	4.0
	6	18.3	9.0
CP-NCP	1	11.5	5.0
	2	11.3	3.3
	3	18.9	6.4
	4	12.0	10.1
	5	12.2	8.1
	6	7.7	5.7
