# Supplementary material for: Human Adaptive Behavior in Common Pool Resource Systems
Source: PLoS One. 2012 Dec 28;7(12):e52763. doi: 10.1371/journal.pone.0052763 (PMC3532302; doi:10.1371/journal.pone.0052763)
Supplement: Text S1 — Additional model results. (PDF) [file pone.0052763.s007.pdf]

Text S1 Additional model results.

The best model results with a variable $w^\star$ and observations made by Janssen et al. (2010) show similar dynamics in all rounds and for all treatments (Figure S2). The biggest deviance between model and observations occurs in the sixth round of the treatment NCP-CP, when the model underestimates resource growth at the beginning of the round. Despite the simplicity of the model and despite a fixed parameter set for all rounds (except for the parameter $w^\star$) the correlation between simulated and observed data is remarkably high. A linear regression confirms this finding with $r^2 = 0.97$ (Figure S3).

The corresponding simulated total harvests (Figure S1) for the different treatments approximate the pay-offs the users realized in the laboratory experiments. It is notable, however, that the spread of the experimental data indicated by the error bars in Figure S1 exceeds 100 N for all treatments with $H_{tot} > 300$ N. Strong variability in the cooperation of users in replicates of this simple experiment is, thus, common and suggests that the influence of individuals in small groups, here consisting of only five people, may be considerable. On the contrary, the model simulates the dynamics of an average group of users realizing an average pay-off and does not account for the variability observed in corresponding rounds with identical treatment in the experiments.

The variation of three parameters ($w^\star$, α, and β) to match the experimental results further improves the model-to-data fit (Figure S4 and Table S1) indicating that though variations in $w^\star$ explain most of the observed differences between rounds, users may also adjust the timing of harvesting from round to round. A systematic sensitivity analysis corroborates the high sensitivity of the model-data fits to changes in $w^\star$, but also indicates that α and β, the two parameters defining the decay of w over time, have an important influence on the correspondence of model results and experimental data.
